# Microfluidic Determination of Distinct Membrane Transport Properties between Lung Adenocarcinoma Cells CL1-0 and CL1-5

**DOI:** 10.3390/bios12040199

**Published:** 2022-03-27

**Authors:** Chiu-Jen Chen, Min-Heng Kao, Noel A. S. Alvarado, Yong-Ming Ye, Hsiu-Yang Tseng

**Affiliations:** Department of Mechanical Engineering, National Taiwan University of Science and Technology, Taipei 106, Taiwan; d10903004@mail.ntust.edu.tw (C.-J.C.); m11003333@mail.ntust.edu.tw (M.-H.K.); m11003812@mail.ntust.edu.tw (N.A.S.A.); m10803338@mail.ntust.edu.tw (Y.-M.Y.)

**Keywords:** microfluidics, non-small-cell lung cancer, cell permeability, lab-on-chip, biomedical applications

## Abstract

The cell membrane permeability of a cell type to water (Lp) and cryoprotective agents (Ps), is the key factor that determines the optimal cooling and mass transportation during cryopreservation. The human lung adenocarcinoma cell line, CL1, has been widely used to study the invasive capabilities or drug resistance of lung cancer cells. Therefore, providing accurate databases of the mass transport properties of this specific cell line can be crucial for facilitating either flexible and optimal preservation, or supply. In this study, utilizing our previously proposed noncontact-based micro-vortex system, we focused on comparing the permeability phenomenon between CL1-0 and its more invasive subline, CL1-5, under several different ambient temperatures. Through the assay procedure, the cells of favor were virtually trapped in a hydrodynamic circulation to provide direct inspection using a high-speed camera, and the images were then processed to achieve the observation of a cell’s volume change with respect to time, and in turn, the permeability. Based on the noncontact nature of our system, we were able to manifest more accurate results than their contact-based counterparts, excluding errors involved in estimating the cell geometry. As the results in this experiment showed, the transport phenomena in the CL1-0 and CL1-5 cell lines are mainly composed of simple diffusion through the lipid bilayer, except for the case where CL1-5 were suspended in the cryoprotective agent (CPA) solution, which also demonstrated higher Ps values. The deviated behavior of CL1-5 might be a consequence of the altered expression of aquaporins and the coupling of a cryoprotective agent and water, and has given a vision on possible studies over these properties, and their potential relationship to invasiveness and metastatic stability of the CL1 cell line.

## 1. Introduction

Lung cancer accounted for 1.8 million deaths in 2020, is currently the leading cause of cancer death around the world, and 85% of all lung cancer cases are non-small-cell lung cancer (NSCLC). With often a late diagnosis, uncontrollable distant metastasis, and anti-cancer drug resistance of the tumor—even if the patients have gone through chemotherapy, surgery, or radiotherapy—the five-year survival rate of lung cancer can be around 10% to 20% or lower [1,2]. The widespread metastasis of lung adenocarcinoma generally occurs in a relatively earlier stage, implying that elucidation of the nature of these cells and an applicable model is fairly important for clinical diagnoses [3]. In 1992, Chu et al. established four different lung adenocarcinoma cell lines, namely CL1, CL2, CL3, and NCL2, to characterize the mucin differentiation. The results demonstrated that the CL1 cells not only displayed no mucin differentiation but also had a higher proliferative index, making CL1 a target to further study on [4]. Later on, in 1997, they proposed a methodology to separate the CL1 cell line depending on invasive and metastasis capabilities, by repeating the incubation-harvest cycle; CL1-0 through CL1-5 was obtained in each 72 h procedure, from 0 to 5 being progressively invasive in vitro and in vivo, characterized by the enhanced expression of 92-kD gelatinase, keratins 8 and 18 [5].

In previous comparative studies and genomic analyses of the CL1-0 and CL1-5 cell lines, the CL1-5 subline was reported to have several attributes that are positively correlated with its invasiveness and metastatic potential [6,7,8,9]. Among these attributes are higher genetic expressions of cytoskeleton and motility proteins, cell cycle regulators and protease, etc., over the parental cell CL1-0. For example, matrix metalloproteinase-2 (MMP-2) and MMP-9 are the major proteases correlated to the invasion and metastasis of lung carcinoma; due to their collagen (the main element constructing the basement membrane)-degrading nature, and the greater expression of MMPs in CL1-5 gives the cancer cell a larger potential to destabilize [10,11,12]. Moreover, the more aggressive cell line also demonstrates a migration phenomenon when subjected to DC (direct current) electric fields (electrotaxis) [13,14,15,16]. Huang et al. displayed a significantly stronger electrotaxis effect of CL1-5 over CL1-0 under different field intensities, which was usually found in highly metastatic cancer cells [17]. Since these characteristics have been discovered, they can now be implemented into assays to serve as targets, developing possible solutions for more thorough clinical diagnoses of the composition of tumors.

Accurate permeability data is the key to optimal cryopreservation. By obtaining the values, we are able to develop preservation protocols suitable for each cell type, and therefore improve the yield of cell culturing, shipping, or stocking conditions of certain precious samples. Substances are able to pass across a cellular plasma membrane through two mechanisms: simple diffusion and channel proteins, such as the aquaporins. By simple diffusion, chemicals are driven by the concentration difference between intracellular and extracellular environments, and the rate of mass transport is proportional to the concentration gradient. On the other hand, channel proteins act as specialized gateways for certain substances, offering an easier (i.e., less activation energy) method for a mass to travel in and out of a cell. Throughout the development of permeability determination methodologies and miscellaneous works featuring either contact- or noncontact-based measurement, direct or indirect observations have been proposed.

The differential scanning calorimetry (DSC), serves as an indirect observation method to derive membrane permeability by calculating the latent-heat generation during the freezing process of the diffused intracellular water. As the data are obtained under cryogenic conditions, DSC is currently the sole configuration to derive the permeability to water at low temperatures [18]. On the downside, DSC is incapable of measuring the permeability to mediums other than water, and its cost of equipment is relatively higher than other available assays, which resulted in difficulties with a wider application. On the other hand, various approaches utilizing direct observation were developed in order to overcome the inconvenience and narrowed usage of DSC. Furthermore, these approaches have built up a comprehensive recognition of the membrane transporting mechanisms. In 1994, Gao et al. first proposed a system that traps mouse oocyte on the tip of a micropipette, and through videotaping, captured the perfusion procedure to obtain the cell volume change, thus permeability [19]. Recently, Weng et al. demonstrated a microarray system featuring a highly occupied, single-cell trapping technique. Upon observing the response of rat hepatocytes and Brx-142 to hyperosmotic solutions, the results manifested the coupling effects in the transport of water and *CPA*s, and the duality of transfer mechanisms (i.e., simple diffusion through the membrane and facilitated diffusion through aquaporins) of the target cells (i.e., rat hepatocytes and Brx-142) [20].

Despite the convenience and intuitive configurations utilized in these assays, other than DSC, the contact-based approaches mentioned above might lead to the problem of confinement geometries that can contribute to unwanted errors. In a sense, to conquer the disadvantages, the development of a noncontact-based system is therefore optimal for solving this problem. In a most recent work addressed by Huang et al., by miniaturizing the electronic particle counter (EPC) and utilizing multiple electrodes, they were able to determine the permeability by measuring the impedance of the cells over time as they flowed through the microchannels [21]. However, the system can be limited to specific uses, as the realistic channel length remains short (a few centimeters for a general lab-on-a-chip device). This can impose inaccuracies in the tests where cells with lower permeability are set as targets, or when the device length was not long enough to support thorough mixing and proper transient profile development, which usually takes minutes to reach equilibrium.

With the lack of determination in permeability properties of this cell line, this study aimed to contribute to the database of CL1, to allow for further studies to utilize accurate references and to provide possible solutions and insights into the knowledge of comparative studies on this specific cell line. In the present study, we implemented a micro-vertex system developed recently by our lab, which is a noncontact-based assay that traps samples inside a swirl to support the direct observation of the full volume-change profile [22,23]. Through this implementation, we were able to free the cell specimens from the geometric confinement, while providing a longer time span for sample observations. Therefore, the determination of plasma membrane permeability can be carried out under the practical assumptions of (1) a fully utilized active osmotic area, and (2) the completely developed volume-change profiles, thus resulting in reasonably accurate values of Lp and Ps.

## 2. Materials & Methods

### 2.1. Theory of Membrane Transport Model

The osmosis between intra- and extracellular environments is driven by the semi-permeable nature of the cell membrane, creating changes in cell volume over time until reaching osmotic equilibrium. The intracellular osmolality under hyperosmotic conditions can be described by the Boyle–van’t Hoff relationship:

(1) $$C_{i}=C_{0}*\left(\frac{V_{0}-V_{b}}{V-V_{b}}\right)$$

where $C_{i}$ is the intracellular osmolality, $C_{0}$ is the extracellular osmolality, V is the cell volume with respect to time, $V_{0}$ is the isotonic cell volume, and $V_{b}$ is the osmotic inactive volume. To evaluate the permeability coefficient to water, consider the volume of a cell in a binary system (i.e., water and a non-permeable solute, such as NaCl) as a function of time, the relationship can be expressed as:

(2) $$\frac{dV}{dt}=L_{p}*A*R*T*\left(C_{i}-C_{e}\right)$$

where $L_{p}$ is the water permeability coefficient, A is the active surface area of the membrane, R is the ideal gas constant, T is the temperature, $C_{i}$ is the intracellular osmolality, and $C_{e}$ is the extracellular osmolality. Since membrane permeability is a temperature-dependent property and can be described by the Arrhenius relationship, we can use its logarithmic form to determine the activation energy, $E_{a}$:

(3) $$L_{p}\left(T\right)=L_{p_{ref}}*exp\left[\frac{-E_{a}}{R}*\left(\frac{1}{T}-\frac{1}{T_{ref}}\right)\right]$$

(4) $$ln[L_{p}\left(T\right)]=\frac{-E_{a}}{R*T}+Constant$$

where R is the ideal gas constant, $L_{p_{ref}}$ is the water membrane permeability coefficient at a known temperature, $T_{ref}$. By calculating the slope of the linear regression line multiplied by R, we can obtain the activation energy required.

Consider the case of a ternary solution system (i.e., water, NaCl, and *CPA*), the net cell volume change according to time can be described by a two-parameter model:

(5) $$\frac{dV}{dt}=\frac{dV_{water}}{dt}+\frac{dV_{CPA}}{dt}$$

in which

(6) $$\frac{dV_{water}}{dt}=L_{p}*A*R*T*\left(C_{i}-C_{e}\right)$$

(7) $$\frac{dV_{CPA}}{dt}=P_{s}*A*\left(C_{e,cpa}-C_{i,cpa}\right)*{\underline{V}}_{CPA}$$

where $P_{s}$ is the cell membrane permeability coefficient to a *CPA*, $C_{e,cpa}$, and $C_{i,cpa}$ are respectively intracellular and extracellular osmolality to the *CPA*, *V_CPA_* is the partial molar volume of the *CPA* in the cell. The $L_{p}$ and $P_{s}$ values can be determined by observing the time rate of cell volume change in the perfusion of a *CPA* solution, and lastly, least-square fitting the data into Equations (5)–(7).

### 2.2. Preparation and Culturing of the CL1 Cell Line

The human lung cancer cell line CL1 was originally derived from a 64-yr-old man suffering from poorly differentiated lung adenocarcinoma and has been passed on for over 60 generations. In this study, the CL1 cells were purchased from the Bioresource Collection and Research Centre (Hsinchu, Taiwan) and cultured with modified RPMI-1640 (HyClone; Cytiva, Marlborough, MA, USA) with 1% HEPES buffer (HyClone; Cytiva, USA), 1% antibiotic antimycotic solution (HyClone; Cytiva, Marlborough, MA, USA), 10% FBS (HyClone; Cytiva, USA) at 37 °C in a humidified incubator (NB-203M, IN-BIOTECH Co. Ltd., Seoul, Korea) with 5% CO2. Cultured cells were washed with 1× phosphate-buffered saline (PBS), then subjected to 0.025% trypsin (HyClone; Cytiva, USA) in order to remove the adhesion tissues. After that, the mixture was centrifuged at 1200 rpm for 5 min. Cell counts were manually conducted using a hemocytometer (Paul Marienfeld GmbH & Co. KG, Lauda-Königshofen, Germany) under the light microscope, and the final concentration of the cell suspension was diluted to 5 × 10^6^ cells/mL.

### 2.3. Experimental Setup, Methods of the Micro-Vortex System and Post-Image-Processing

**Figure 1:**
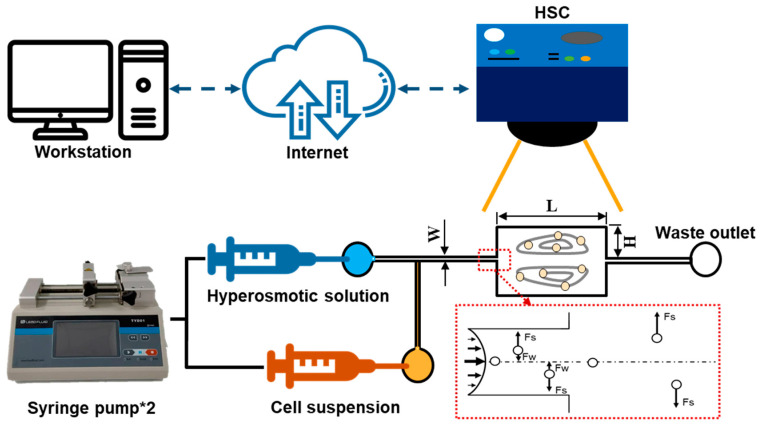
Schematics used for direct observation of the cells trapped in the micro-vortex system to derive the transient volume change profile. The PDMS microchannel has the featured geometries: W: 30 μm, H: 300 μm, L: 900 μm and thickness: 50 μm.

In this work, CL1-0 and CL1-5 were captured by the hydrodynamic circulation formed by the liquid flow in a microfluidic chamber fabricated through standard soft lithographic rapid prototyping of SU-8 (SQ-50, Kemlab Co. Ltd., Woburn, MA, USA) and replica molding with polydimethylsiloxane (PDMS) (DC-184 Elastomer kit, Dow Chemical, Midland, MI, USA). The PDMS microchannel featuring the geometry, demonstrated in Figure 1, was bonded to a glass slide (DG75001-07101, Dogger Scientific Co. Ltd., Taipei, Taiwan) using oxygen plasma (PDC-001HP, Harrick Plasma Co. Ltd., Ithaca, NY, USA) before the experiments. After the channel was produced, it was assembled to form the experimental setup shown, with the combination of two syringe pumps (TYD01, Baoding Lead Fluid Technology Co. Ltd., Baoding, China), a high-speed camera (HSC) (SC1, Edgertronic, San Jose, CA, USA), and an inverted microscope (WI-400, Whited Co. Ltd., Tainan, Taiwan).

As manifested in Figure 1, the CL1 cell lines suspension solution, isosmotic and hyperosmotic solution were injected into a 30 μm width microchannel; cells were trapped inside the expansion region with a channel height of 300 μm and a length of 900 μm, and all the channel thicknesses were controlled by a SU-8 coating with a thickness of 50 μm. The particles flowing into the channel were subjected to a shear-gradient lift (Fs) and an opposite wall-effect lift (Fw). When the cells entered the expansion region, Fw suddenly disappeared, thus, shifting them upwards/downwards into the vortices. As the targeted cells were introduced into the vortices generated in the rectangular chamber (i.e., expansion region), the hypertonic solutions were instantaneously injected into the system to induce cell volume change, which was simultaneously recorded by the high-speed camera for post-processing. The hyperosmotic solutions selected in this study were (1) a 3× PBS solution, and (2) a 10% (*v*/*v*) dimethyl sulfoxide (DMSO) in 1× PBS solution, where DMSO is a commonly used *CPA* for cell preservation. The solutions were implemented into the assay to derive the permeability properties of water and cryoprotective agent, respectively. Note, that as the CL1 cell lines were injected into the microchannel, they were first trapped inside the vortices formed by the isotonic 1× PBS solution. After the hyperosmotic solutions were introduced, a prompt (around 2 s) reagent replacement could be established based on former results and experiences. In addition, since the relatively lower Reynolds number (Re = 125) and small channel diameter featured in this system, mixing phenomena were insignificant between the cell suspension and hyperosmotic solutions; therefore, practical assumptions were made to ignore the above effects, making sure that the perfusion process began after the cells were induced into the micro-vortex, giving a more errorless observation of the mass transport phenomenon [22]. Finally, in order to obtain the temperature dependence of the cell membrane permeability, the operation of this system was conducted under controlled ambient temperatures of 22, 30, and 37 °C using a commercial air conditioner (Panasonic), and the validity of the temperature values was checked using another thermometer.

For image processing, the collection of 24-bit color photos captured by the high-speed camera (exposure time: 50 μs) in the observed region was first turned into 8-bit grayscale images, and the edge of every individual cell was then amplified and plotted by the canny edge detection, in which parameters, such as the aperture size, sigma, and high and low thresholds, were empirically fine-tuned for optimal graphing results. Furthermore, the images were filtered through the basic and advanced morphology operations; the basic operation repaired discontinuities at the edges and helped to form closed objects, while the advanced function filled the objects with pixels and removed critical particles that were either oversized or undersized, which represented cell clusters or cell debris, respectively. Subsequently, for adherent cells, such as CL1 in this study, their volume became sphere-like when suspended in the solutions, thus it would also be practical to analyze the cells using a spherical model during image processing. Afterward, with the assumptions above, the actual cell volume was evaluated based on pixel counts of cell objects. In the end, thousands of processed images were used in the determination of cell volume change with respect to time, which was solved by a mathematical model utilizing MATLAB’s curve-fitting tool, and therefore the permeability coefficients.

## 3. Results and Discussions

### 3.1. Determining V_b_

**Figure 2:**
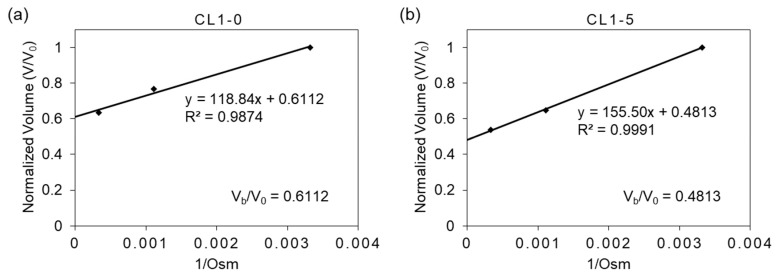
The Boyle–van’t Hoff relationship of (**a**) CL1-0 and (**b**) CL1-5. The equilibrium cell volumes in isotonic (1× PBS) and hypertonic (3× and 10× PBS) saline solutions, normalized to the cell volume in isotonic solution are plotted against the reciprocal of the osmolality of the solution.

The osmotic inactive cell volume (V_b_), composed of the overall volume of cell organelles and bounded water in the cytoplasm, can be determined by the evaluation of cell volume at a theoretical zero osmolality (i.e., the condition at which the cell has nothing to transport to a hypertonic solution); this was done through the linear regression of the Boyle–van’t Hoff relationship shown in Figure 2, where the _y_-axis intercept indicates the ratio between inactive volume (V_b_) to the isotonic volume (V_0_). As V_b_ is considered irrelevant to the determination of cell membrane permeability, it was subtracted from the cell volume V in Equation (1). In the cases of this study, V_b_/V_0_ is estimated to be 0.6112 and 0.4813 for CL1-0 and CL1-5, respectively.

### 3.2. Determining Lp of CL1-0 & CL1-5 in the Binary System

**Figure 3:**
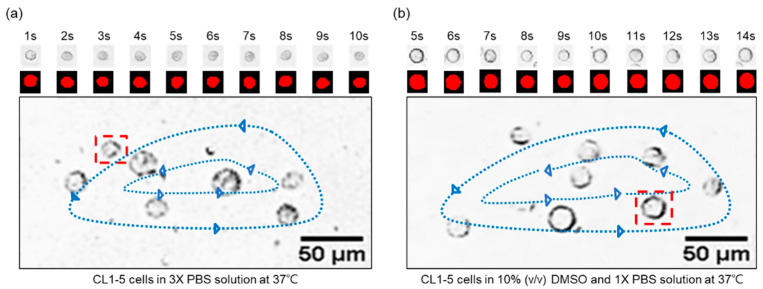
The CL1-5 transient volume change from the footages of the cells circulating in a vortex of (**a**) 3× PBS solution, and (**b**) 10% (*v*/*v*) DMSO and 1× PBS solution. The ambient temperature is controlled at 37 °C, and a one-second interval is selected to generate a sequence of images that demonstrate the trend of volume variations. Please note that the sequential images shown in the figure present only the selected part of the recorded video for demonstration purposes.

As depicted in Figure 3, the transient volume change of circulating CL1-5 cells in the vortex can be observed through footages from the HSC, and with the grayscaled- and morphology-modified photos presented. The volume-change profiles can then be generated by counting the red pixels. Please note that the cells may not always maintain 100% sphericity during the experiments, leading to deviation from our perfect-sphere assumption when calculating the membrane permeability. We would also be very interested in measuring the three-dimensional profile of CL1 cells to retrieve their actual sphericity, yet the technology (confocal microscopy can only be applied to immobilized or adherent cells with inevitable deformation) is unfortunately not available or accessible to us for streaming cells. From direct observation under the microscope, the almost-perfect-round shape of CL1 cells was subjectively observed by us. However, we are not practically able to find a proper way to contour and demonstrate the true shape of CL1 cells and to support our assumption or adjust our calculation accordingly. In general, cells in suspension tend to maintain a spherical shape, except for well-known donut-like red blood cells; the perfect-sphere assumption has therefore been accepted by the field for calculating cell membrane permeability, while we acknowledge that the potential influences of cell shape on the calculation would be an important and necessary topic for further investigation.

**Figure 4:**
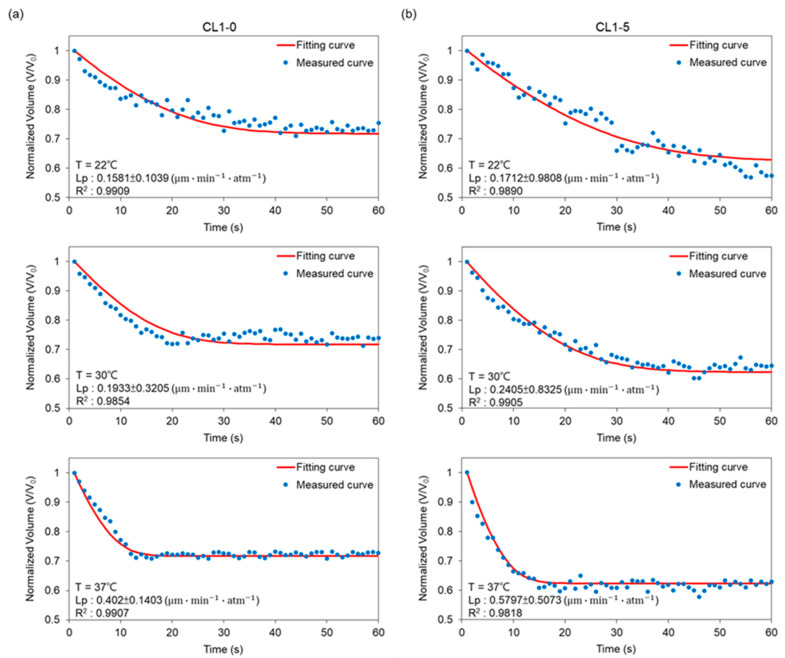
Volumetric changes of (**a**) CL1-0 and (**b**) CL1-5 under exposure to 3× PBS at 22 °C, 30 °C, and 37 °C, respectively. The predicted curve was calculated according to Equation (2).

Plotted in Figure 4 are respectively the transient profiles of normalized-volume change through time for CL1-0 and CL1-5 in the binary system (i.e., NaCl-water). Measured points were curve-fitted in MATLAB to empirically derive Lp from equation (2), which were respectively 0.16, 0.19, and 0.40 $\mu m\cdot \min^{-1}\cdot {\mathrm{atm}}^{-1}$ for CL1-0; 0.17, 0.24, 0.58 $\mu m\cdot \min^{-1}\cdot {\mathrm{atm}}^{-1}$ for CL1-5 at 22, 30, and 37 °C. The outcomes were then plugged into equation (2) to obtain the predicted curves, and by regression, presented in terms of $R^{2}$, exhibiting the fact that our data points well fit the predictions. As manifested in Figure 3, the volume shrinkage was about 30% at each tested temperature when reaching osmotic equilibrium, with results at 37 °C having a more abrupt volume drop than those at 22 and 30 °C. Moreover, the data also showed a decrease in time reaching a steady state when the ambient temperature was raised. This suggested that the cell membrane permeability increases as a response to temperature elevation, and especially under a moderate condition such as the human body temperature, 37 °C, a non-proportional boost in its Lp values can be triggered.

In comparison to CL1-0, the results for the CL1-5 subline in Figure 4b demonstrated not only a 40% volume shrinkage during the osmosis process but also a later establishment of equilibrium at 22 and 30 °C. However, surprisingly, the transport phenomena at 37 °C resemble its CL1-0 counterpart, which again showed significantly higher permeability driven under this temperature, and further, an identical transition interval from transient to steady states. Combining the above features suggests that the membrane kinetics of the CL1 cell line suppressed under conditions deviates from the human body or tumor microenvironment.

### 3.3. Solving for Lp and Ps in the Ternary System

**Figure 5:**
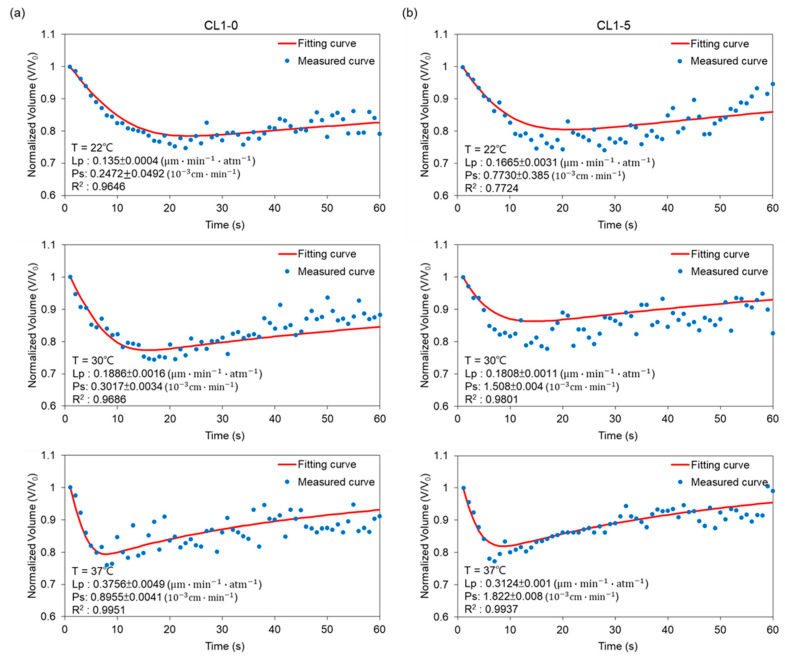
Volumetric changes of (**a**) CL1-0 and (**b**) CL1-5 suspended in a solution containing 10% DMSO and 0.9% NaCl (**a**,**b**) at 22, 30, and 37 °C, respectively. The predicted curve was calculated according to Equations (6) and (7).

Applying a similar manner discussed in the previous section, the targeted cells were perfused with the cryoprotective agent solution to form a ternary system, and to determine the permeability of the CL1 cell line with the presence of *CPA*. As shown in Figure 5, cells trapped inside the ternary system first experienced a volumetric shrinkage caused by the hyperosmotic extracellular environment, generating an outflux of water from the intra- to extracellular region. Subsequently, when the specimens had shrunk to 80% of their original volume, the cell-penetrating *CPA* influx started to take dominance, which pushed the volume back to its isotonic value. To sum up the results: the Lp values for the CL1-0 cell line are 0.14, 0.19, and 0.38 $\mu m\cdot \min^{-1}\cdot {\mathrm{atm}}^{-1}$; Ps are 0.25 × 10^−3^, 0.30 × 10^−3^, and 0.90 × 10^−3^ c$m\cdot \min^{-1}$, at 22, 30, and 37 °C, respectively. As for the CL1-5 counterpart, Lp is 0.17, 0.18, and 0.31 $\mu m\cdot \min^{-1}\cdot {\mathrm{atm}}^{-1}$; 0.77 × 10^−3^, 1.51 × 10^−3^, and 1.82 × 10^−3^ $\mathrm{cm}\cdot \min^{-1}$ for Ps, corresponding to temperatures of 22, 30, and 37 °C.

Through observing Figure 5, we can see that Lp and Ps in this ternary system both demonstrated positive temperature dependencies, with the permeability at 37 °C having non-proportional increases. Focusing on the behavioral differences between CL1-0 and CL1-5; within a DMSO introduced extracellular environment, the Lp values for CL1-0 at the tested temperatures did not deviate a lot from those obtained in the binary system, while in the case of CL1-5, under 37 °C, the water permeability coefficient dropped from 0.58 to 0.31 $\mu m\cdot \min^{-1}\cdot {\mathrm{atm}}^{-1}$. The presented data imply that the *CPA*’s existence suppressed the fluctuation of water transfer by occupying active areas on the membrane, or that it inhibited the channel diffusion through aquaporins, causing an overall decrease in water flux through CL1-5 cells. On the contrary, CL1-0 seems to be able to fully facilitate osmosis and diffusion even under the existence of DMSO, suggesting that *CPA* transfer does not use up a considerable amount of active area, or that the phenotypes of aquaporins are resistant to DMSO and can still function normally.

### 3.4. Temperature-Dependent Osmosis of the CL1 Cell Line

**Figure 6:**
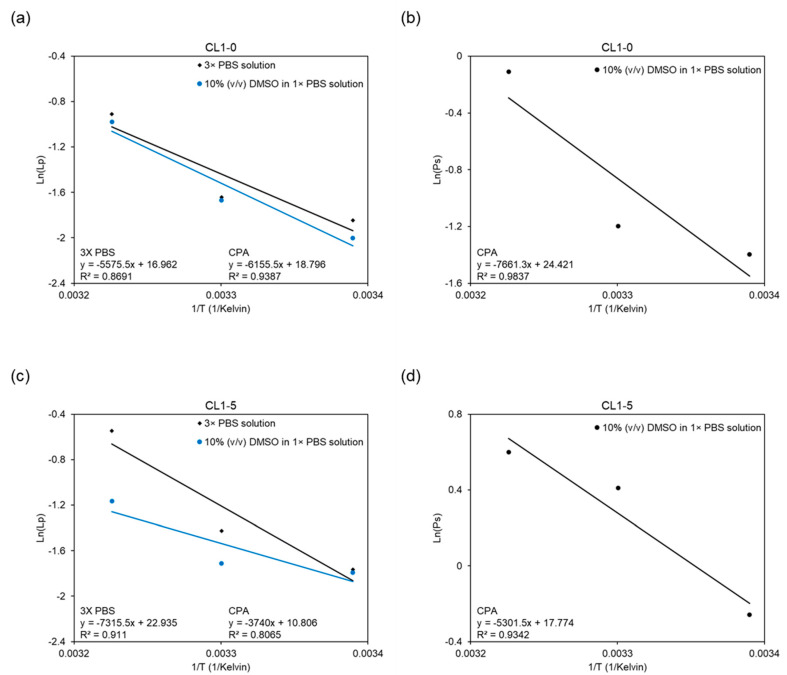
Arrhenius diagram showing the natural logarithms of the average membrane permeability of CL1-0 and CL1-5 versus the reciprocal of the absolute temperature for (**a**,**c**) Lp in different hyperosmotic solutions, and for (**b**,**d**) Ps in the ternary system.

Utilizing the logarithmic form of the Arrhenius relation (Equation (4)), the data were fitted into the equation by linear regression, as demonstrated in Figure 6. The activation energy Ea serves as an indicator of the temperature dependency of membrane permeability in this study. Shown below in the graphs, the slopes of the linear regression lines represent values of -Ea/R in Equation (4); the more negative the slopes are, the higher the activation energy, and therefore greater are the temperature dependencies. In the CL1-0 case, Ea values were derived to be 11.08 kcal/mol in the binary system, while in the ternary system, 12.23 kcal/mol for Lp and 15.22 kcal/mol for Ps were obtained. In comparison, the CL1-5 required 14.54, 7.43, and 10.54 kcal/mol of activation energies for, respectively, the Lp in binary, Lp in ternary, and Ps in ternary setups. In addition, the change of slope (i.e., activation energy) when cell specimens were subjected to different systems also displayed opposite behaviors. While the CL1-0 cell line manifested a slight and negative deviation in slope, demonstrated in Figure 6a, CL1-5 conversely showed a significant yet positive change in Figure 6c.

### 3.5. Discussions

Through the results shown in Table 1, we can see, first of all, the obvious temperature dependencies where Lp and Ps rose with elevated temperatures, and secondly, the variation in activation energy of Lp when cells were in different systems. Based on previous studies, for Ea over a cut-off value of 10 kcal/mol, the water transport phenomena were reported to be dominated by simple diffusion through the lipid bilayer; on the other hand, for cells possessing activation energies of less than 6 kcal/mol, the overall effect was contributed by the aquaporin channels [24]. Given in the data, Ea values for Lp and Ps are generally over a threshold of 10 kcal/mol except for the case for Lp of CL1-5 in a ternary system, which is only 7.43 kcal/mol. The above facts thus might suggest that (1) the simple diffusion mechanism for a CL1 cell line to water or DMSO has a general dominance over osmosis facilitated by aquaporin channels, and (2) DMSO makes the water channels become more involved in the transfer operation, resulting in the lowest Ea value in all data. Recent studies have already demonstrated the altering of aquaporin expressions on lung cancer cell lines A549 and CL1-5 as responses to increased hydrostatic pressure, which ended up promoting cell migration [25,26]. In addition, several research studies focused on different NSCLC cell lines, such as the H1299, XWLC-05, which have also given a disclosure on the close correlations between aquaporin expressions and metastatic potentials [27,28,29,30,31,32]. Therefore, the data presented in this study might also show that CL1-5 cells possess similar properties responding to extracellular environment change other than hydrostatic pressure, and this requires extended research on the CL1 cell line in order to build a profound recognition of this phenomenon.

## 4. Conclusions

In this work, a comparative study on the difference in membrane permeability (Lp & Ps) between the human lung adenocarcinoma cells, CL1-0 and CL1-5, was conducted through a micro-vortex system, previously developed by our group, featuring direct observation and noncontact-based sampling. The CL1 cells were confined in a hydrodynamic circulation while the volumetric change with respect to time was recorded by a high-speed camera, which contributed to measurement without physical confinement and instant generation of a well-distributed osmotic gradient. The theme of this work aimed to offer accurate values of the membrane permeability coefficients, which currently lack the data, so as to optimize cryopreservation protocols. On the other hand, calculated values of the membrane-transport properties also presented the nature behind these specimens, providing us with the chance to tackle fatal diseases and construct well-rounded recognitions. With CL1-0 being the parental cell, and CL1-5 being its more metastatic subline, the two specimens demonstrated different behaviors under three distinct ambient temperatures. First, the cells presented a boost in permeability when exposed to the surroundings of 37 °C, expressing a positive dependence between elevated temperature and membrane transportation, and the difference in performance as the cells were subjected to an optimal environment. In addition, comparing the volume profiles from Figure 4, CL1-5 reached equilibrium in a generally later manner in the binary system with a higher flux at 22 and 30 °C; however, at 37 °C, CL1-5 developed a profile similar to the CL1-0 counterpart. This surprisingly implies that the membrane kinetics of the CL1 cell line might be suppressed under undesired conditions. Second, the results showed that for the parental cell line CL1-0, Lp values did not deviate much from each other in the binary and ternary system, which implied that with the introduction of DMSO, it only had a slight influence on the water permeability of CL1-0. This might be a consequence of DMSO occupying active surface areas, causing the water flux to decrease. On the contrary, CL1-5 displayed not only a larger drop in Lp but also a nearly halved activation energy when suspended in the *CPA* solution. The obtained data suggest that the aquaporin expressions of these more aggressive CL1 subline membranes might contribute to shifts in the dominant diffusion mechanism, thus promoting chances of metastasis and proliferation. Furthermore, as manifested in Table 1, the Ps values for CL1-5 are greater than those of CL1-0. Combined with a much lower Ea of 7.34 kcal/mol for Lp of CL1-5, it might be reasonable to draw the conclusion that DMSO activated the aquaporins and occupied the active osmotic area of a cell, triggering the generally higher results in Ps, while keeping Lp at relatively low values. Requiring extensive research in the future, these differentiated expressions of water channel proteins have the potential to become biomarkers targeted by chemotherapies or assays, which can help to implement more precise diagnoses and curing methods for clinical purposes, disclosing a new fragment in the complex mechanisms behind cancer invasion and metastasis.

## Figures and Tables

**Table 1:** Lp, Ps, and Ea of CL1-0 & CL1-5 cells at various temperatures (mean ± standard deviation).

CL1-0	Temperature (°C)	Lp ± SD (n, r^2^) $\left(\mu m\cdot min^{-1}\cdot atm^{-1}\right)$	Ea of Lp (r^2^) (Kcal/mol)	Ps ± SD (n, r^2^) $\left(10^{-3}\;cm\cdot min^{-1}\right)$	Ea of Ps (r^2^) (Kcal/mol)
PBS-Water	22	0.16 ± 0.10 (10, 0.99)	11.08 (0.87)	-	-
	30	0.19 ± 0.32 (7, 0.99)		-	
	37	0.40 ± 0.14 (10, 0.99)		-	
PBS-Water-DMSO	22	0.14 ± 0.01 (14, 0.96)	12.23 (0.94)	0.25 ± 0.05 (14, 0.89)	15.22 (0.98)
	30	0.19 ± 0.01 (12, 0.75)		0.30 ± 0.01 (12, 0.97)	
	37	0.38 ± 0.01 (8, 0.82)		0.90 ± 0.01 (8, 0.99)	
**CL1-5**	**Temperature (°C)**	**Lp ± SD (n, r^2^)** **(** $\mu m\cdot min^{-1}\cdot atm^{-1}$ **)**	**Ea of Lp (r^2^)** **(Kcal/mol)**	**Ps ± SD (n, r^2^)** **(** $10^{-3}\;cm\cdot min^{-1}$ **)**	**Ea of Ps (r^2^)** **(Kcal/mol)**
PBS-Water	22	0.17 ± 0.98 (17, 0.99)	14.54 (0.91)	-	-
	30	0.24 ± 0.83 (18, 0.99)		-	
	37	0.58 ± 0.51 (4, 0.98)		-	
PBS-Water-DMSO	22	0.17 ± 0.01 (11, 0.77)	7.43 (0.81)	0.77 ± 0.39 (11, 0.77)	10.54 (0.93)
	30	0.18 ± 0.01 (20, 0.83)		1.51 ± 0.01 (20, 0.98)	
	37	0.31 ± 0.01 (4, 0.99)		1.82 ± 0.01 (4, 0.99)	

## Data Availability

The datasets used and/or analyzed for the present work are available upon request.
